# Editorial: Psychological Distress Among University Students

**DOI:** 10.3389/fpsyg.2021.647940

**Published:** 2021-03-22

**Authors:** Antonella Granieri, Isabella G. Franzoi, Man C. Chung

**Affiliations:** ^1^Department of Psychology, University of Turin, Turin, Italy; ^2^Department of Educational Psychology, Faculty of Education, The Chinese University of Hong Kong, Shatin, Hong Kong

**Keywords:** university students, emerging adulthood, distress, interventions, measures

Emerging adulthood (Arnett, 2000, Arnett et al., 2014) is a developmental phase between adolescence and adulthood characterized by many transitions and challenging tasks, such as financial self-sufficiency, choices about career and intimate relationships, and preparing the ground for adult lives (Furnham, 2004; Miller, 2017; Schechter et al., 2018).

For some emerging adults, this phase coincides years of higher education, which implies further tasks: performance demands, changes in living conditions, dealing with a new social and educational context (Settersten and Ray, 2010; Schulenberg and Schoon, 2012). Starting higher education seems to constitute a crucial transition for mental health (Molina et al., 2012; Pedrelli et al., 2015; Auerbach et al., 2016; Harris, 2019), since students consistently report higher levels of distress compared to the general population (James et al., 2017; Tariku et al., 2017; Mboya et al., 2020). In particular, university students seem characterized by high levels of depression, anxiety and suicide risk (see among others Beiter et al., 2015; Auerbach et al., 2016; Larcombe et al., 2016; Rotenstein et al., 2016; Oyekcin et al., 2017; Poorolajal et al., 2017; Tran et al., 2017; Villatte et al., 2017; Tang et al., 2018). Besides, trauma (e.g., childhood abuse, accident, or assault) is a common occurrence, with a percentage ranging from 52 to 85%, of whom 9 to 59% of students might risk developing posttraumatic stress disorder.

University students' distress seems to be particularly influenced by the peer context to which students are exposed on a daily basis (Collins and Laursen, 2004). In particular, peer support seems to protect students' mental health (Alsubaie et al., 2019): indeed, those who report higher levels of perceived social support, also report lower levels of all types of loneliness (Bernardon et al., 2011). Besides, university students' well-being can be also influenced by peer pressure (Alsubaie et al., 2019), that seems to have a role in increasing alcohol and drug consumption (Knee and Neighbors, 2002; Monaci et al., 2013; Abikoye et al., 2014), body image concerns (Webb and Zimmer-Gembeck, 2014), and internet addiction (Esen and Gündogdu, 2010). At the same time, family emotional support seems to have a great importance on happiness and life satisfaction (North et al., 2008; Delle Fave et al., 2010; Hsu, 2010). However, recent literature evidenced that with the transition to university, the importance of peer support becomes more influential and critical than family support (Alsubaie et al., 2019; Kim, 2020), probably because university students have more interactions and similar experiences to their peers than their family (Bernardon et al., 2011).

In these times, we have also to consider the impact of the novel coronavirus disease of 2019 (Covid-19) pandemic, that aroused fears and anxiety globally, leading to an upsurge in the incidence and severity of mental health problems (Granieri et al., 2020; Serafini et al., 2020; Xiong et al., 2020). Indeed, covid-19 pandemic caused a massive interruption in the lives and education of university students worldwide, entailing prolonged school closure, transition to internet-based learning, and social isolation from peers during state-enforced quarantine (Bourion-Bédès et al., 2021; Sun et al., 2021). In particular, worries, isolation from social networks, a lack of interaction and emotional support from friends, as well as physical isolation were associated with negative mental health trajectories among university students (Husky et al., 2020; Meda et al., 2021). Lukács (2021) evidenced that university students experienced significant negative changes after the first month of isolation regarding physical activity, relationship with family and friends, financial situation, perceived health, and perspective for the future. Morever, age, housing conditions, previous history of mental issues, anxiety, and fear significantly predicted psychological distress (Saravanan et al., 2020). Moreover, stress related to academics (e.g., personal uncertainties about academic program, distance teaching), health, and lack of social support predicted more negative mental health impacts (Lai et al., 2020).

These data are matters of particular interest for mental health and educational systems worldwide. Indeed, students experiencing higher psychological distress are at a higher risk of academic failures and dropout (Jaisoorya et al., 2017; Ishii et al., 2018), with important implications for campus health services and mental health policymaking (Viñas Poch et al., 2004).

These considerations are supported by the 13 articles which were included in the Research Topic “Psychological Distress among University Students.”

Focusing on instrument development and evaluation, May, Terman et al. reported on the initial development and validation of the eight-item Burnout Stigma Instrument (BSI-8). First, they described the construction and initial evaluation of scale items, and they replicated in a second study the factor structure determined in the initial study. Resulted of earlier exploratory and confirmatory factor analyses were replicated using item response theory. Moreover, they found out that burnout stigma predicted mental health indicators 6 weeks later. In another paper, May, Rivera et al. reported both latent profile and item response theory analyses of the School Burnout Inventory in US undergraduate students. Latent profile analysis identified four mutually exclusive subgroups based on patterns of school burnout responses, that were linked to meaningful indicators of academic success and health. Longitudinal analysis suggested that these profiles followed a relatively stable trajectory over time. Moreover, the item response theory analysis identified a more concise four-item scale.

Focusing on distress,  Hernández-Torrano et al. used bibliometric procedures to describe literature on mental health and well-being in university students, using metadata extracted from articles dated 1975–2020. They found out that research has grown over the last decades, was disseminated in a wide range of journals, mainly psychology, psychiatry, and education research ones, was published by researchers with diverse geographical background, and by a number of research groups scarcely in contact between them. They also found out that published papers were relatively interdisciplinary, tended to emphasize pathogenic approaches to mental health, and mainly focused on positive mental health, mental disorders, substance abuse, counseling, stigma, stress, and mental health measurement. Furthermore,  Karyotaki et al. investigated perceived stress across major life areas in college students from 24 universities in nine countries. They found out that he 93.7% of students reported at least some stress in at least one area. Besides,  Franzoi et al. found out concerning levels of distress in students at an Italian University. 44.1% of students showed state anxiety, 61.6% showed trait anxiety, while they showed moderate to severe depression in 20.4% of cases. Moreover, Backhaus et al. conducted a cross-sectional study among students at their first year at university in Europe, Asia, the Western Pacific, and Latin and North America. 48% of students presented clinically relevant depressive symptoms, with considerably high rates among students from Brazil (86%). Focusing on impostor feelings,  Fassl et al. found out a highly prevalence of these feelings in a sample of Austrian university students: more specifically, 8.6% experienced few, 40.3% moderate, 38.5% frequent, and 12.6% intense impostor feelings. They also showed that the impostor phenomenon had a moderate and negative relationship with positive masculinity, and a strong and positive correlation with negative aspects of femininity. Moreover, the relationship between impostor feelings and negative femininity was partially mediated by social comparison orientation. Finally,  Wang and Zhao investigated anxiety in a sample of Chinese university students after the outbreak of Covid-19 right before the start of new spring term. 15.43% of students screened positive for anxiety. Interestingly, no significant correlation was found between students' anxiety and affected cases of Covid-19 in their cities or provinces.

For what concerns studies investigating associations between distress and other variables, Karyotaki et al. found out associations between stress in all areas except for health and well-being of loved ones and mental disorders, as well as a significant dose-response association between extent of stress in each life area and increased odds of at least one mental disorder. Moreover, the multivariable models that included all stress measures were significant for all disorders Moeller et al. found out that stress management techniques were related both to students' maladaptation and adaptation in a sample of US undergraduate students. Moreover, Franzoi et al. found a significant positive effect of negative affectivity on alexithymia, and that alexithymia was a mediator between negative affectivity and both state and trait anxiety, while controlling for age and gender. However, their results did not confirm the impact of students' housing conditions on anxiety. Besides, Moeller et al. (2020) found out that belongingness partially mediated the association between emotional intelligence and adaptation in a sample of US undergraduate students. Students with stronger emotional intelligence showed higher levels of belongingness which, in turn, was associated with better overall mental health. Conversely, students with lower emotional intelligence showed higher levels of rejection which was linked to higher depression, anxiety, and distress. Emotional intelligence was considered also by Navarro-Mateu et al. Emotional clarity and self-efficacy were negatively related to stress and positively related to emotional attention, while none of the variables was a necessary condition for inducing stress, even if the interaction between high levels of emotional attention and low levels of self-efficacy was significant for high levels of stress, and low levels of perceived stress seemed connected to the interaction between low levels of self-efficacy and low levels of emotional attention. Besides, Backhaus et al. found out that lower levels of individual and macrolevel social capital were significantly associated with clinically relevant depressive symptoms among university students. The likelihood of showing depressive symptoms was also significantly higher among students living in regions with lower levels of social capital.

These results underline that it is of fundamental importance to adopt an integrated approach toward university students' psychological distress.  Franzoi et al. suggested the need to develop preventative and therapeutic interventions tailored to students' clinical characteristics, Moeller et al. (2020) suggested to target interventions to students experiencing greater feelings of rejection or isolation, while  Navarro-Mateu et al. suggested the promotion of measures and intervention programs aimed at improving students' quality of life, and Backhaus et al. suggested the implementation of on-campus mental health counseling services and interventions focused on enhancing social capital. Moreover, Karyotaki et al. suggested that up to 46.9–80.0% of 12-month disorder prevalence might be eliminated if stress prevention interventions were developed. Besides, Binder et al. found out that even a short intervention might be sufficient to facilitate positive change in non-clinical student samples. Their results showed that students started treating themselves better in their everyday life. They became more supportive and friendlier toward themselves, as well as more aware of how harshly they treated themselves in difficult situations. Moreover, they experienced painful emotions in different ways, finding relief in considering suffering as part of the human condition, and accepting uncomfortable feelings. Consequently, they started feeling more stable and peaceful, and capable to face pressures and challenges connected to their academic and personal lives. Finally, Frewen et al. evaluated an unguided “safe-place” imagery task, a narrative episodic recall task, and a guided wilderness imagery task comparing a virtual reality condition, a 2-D condition, or an eyes-closed mental imagery condition. The virtual reality condition showed the greater participants' perceived satisfaction and credibility, and the greatest positive affect in response to the intervention.

These results underline that is crucial that more comprehensive services are provided to support students with mental health concerns. Currently, though, mental health services give priority to first-level interventions (Conley et al., 2015). However, it would be of great importance to increase the provision of psychotherapy and psychiatric intervention, thereby addressing a significant public health problem among emerging adults (Cleary et al., 2011).

## Author Contributions

All authors equally contributed to the manuscript and approve the final version of the paper to be published and agree to be accountable for all aspects of the work in ensuring that questions related to the accuracy or integrity of any part of the work are appropriately investigated and resolved.

## Conflict of Interest

The authors declare that the research was conducted in the absence of any commercial or financial relationships that could be construed as a potential conflict of interest.
